# Effect of SGLT-2 inhibitors on liver fibrosis progression in patients with MASLD: an updated meta-analysis based on RCTs

**DOI:** 10.3389/fmed.2025.1667823

**Published:** 2026-01-20

**Authors:** Lei Yang, Jiale Ruan, Yingying Fang, Anyi Xu

**Affiliations:** 1 Emergency Medical Center, Ningbo Hospital of Integrated Traditional Chinese and Western Medicine, Ningbo, Zhejiang, China; 2 The First Affiliated Hospital of Zhejiang Chinese Medical University (Zhejiang Provincial Hospital of Chinese Medicine), Hangzhou, Zhejiang, China

**Keywords:** liver fibrosis, meta-analysis, metabolic dysfunction-associated fatty liver disease, nonalcoholic fatty liver disease, SGLT-2 inhibitors

## Abstract

**Background:**

The purpose of our study was to assess the effect of sodium–glucose cotransporter protein 2 (SGLT-2) inhibitors on the progression of liver fibrosis in patients with non-alcoholic fatty liver disease (NAFLD), which is currently renamed metabolic dysfunction-associated steatohepatitis (MASLD).

**Methods:**

From database establishment to February 2025, we systematically searched electronic databases, including PubMed, Web of Science, Embase, and the Cochrane Library, to identify relevant randomized controlled trials (RCTs). The mean difference (MD) and 95% confidence intervals (CIs) were used to assess the effects of SGLT-2 inhibitors on liver fibrosis indicators, including the Fib-4 index, NAFLD fibrosis score (NFS), liver stiffness measurement (LSM), controlled attenuation parameter (CAP), and serum type 4 collagen 7s levels.

**Results:**

A total of 16 RCTs involving 11,300 subjects were included. The meta-analysis revealed that, compared with the control group, SGLT-2 inhibitors significantly reduced the Fib-4 index (MD = −0.16, 95% CI: −0.32 to 0.00, *p* = 0.05), NFS (MD = −0.10, 95% CI: −0.16 to −0.04, *p* = 0.01) and serum type 4 collagen 7s levels (MD = −0.35, 95% CI: −0.63 to −0.06, *p* = 0.02) in NAFLD patients. However, no significant differences were observed in imaging metrics such as LSM and CAP. Subgroup analyses indicated that empagliflozin and ipragliflozin may be more efficacious, with their benefits more pronounced in patients receiving short-term treatment (<24 weeks) and those with combined T2DM.

**Conclusion:**

SGLT-2 inhibitors may delay the progression of liver fibrosis in patients with MASLD, particularly by improving serologic parameters. However, additional high-quality studies are needed to validate their clinical value.

## Introduction

Non-alcoholic fatty liver disease (NAFLD) has become a major global health problem, affecting approximately 38% of the world’s population, with projections suggesting an increase to 55% by 2040 (1). NAFLD is characterized by excessive fat accumulation in the liver, associated with insulin resistance (IR) and evidence of steatosis based on imaging or histology. Secondary causes of hepatic steatosis, such as excessive alcohol consumption (>30 g/day in men and >20 g/day in women), must be excluded (2). Obesity and type 2 diabetes mellitus (T2DM) are strong risk factors for NAFLD and are closely linked to its progression. Additionally, NAFLD, as a chronic liver disease, is associated with an increased risk of cardiovascular disease, chronic kidney disease, cirrhosis, and hepatocellular carcinoma (1, 3). In 2023, NAFLD was officially renamed “metabolic dysfunction-associated steatohepatitis” (MASLD) to better reflect its pathophysiology. This change is not merely a name change but reflects a more accurate understanding of the disease’s core “metabolic” pathogenesis. The key difference between the former and updated definitions lies in the diagnostic criteria. The diagnosis of NAFLD is based on exclusion criteria (“excluding other causes”), whereas the diagnosis of MASLD relies on positive criteria—particularly, the presence of hepatic steatosis accompanied by at least one cardiometabolic risk factor (such as overweight/obesity, type 2 diabetes, and hypertension). Consequently, the MASLD definition is more inclusive and clinically actionable (4–6).

Sodium–glucose cotransporter-2 (SGLT-2) inhibitors, a class of hypoglycemic drugs used to treat T2DM, have shown promise in managing MASLD (7–9). However, their roles in mitigating liver fibrosis, a long-term adverse outcome of MASLD, remain to be fully elucidated.

A previous meta-analysis by Ong Lopez et al. (10) reported that SGLT-2 inhibitors slightly improved hepatic fibrosis in NAFLD, but the results were highly heterogeneous, and subgroup analyses were not performed. To address these limitations, we conducted an updated meta-analysis to explore the effects of SGLT-2 inhibitors on liver fibrosis progression in patients with MASLD.

## Method

This meta-analysis adhered to the Preferred Reporting Items for Systematic Reviews and Meta-Analyses (PRISMA) guidelines (11) and was registered on PROSPERO (CRD420251008470).

### Search strategy

From database inception to February 2025, we searched PubMed, Web of Science, Embase, and the Cochrane Library using terms related to SGLT-2 inhibitors and MASLD. Two researchers independently conducted the search and manually screened the reference lists of relevant articles to identify additional studies. The search terms included the following terms: (“Sodium-Glucose Transporter 2 Inhibitors”[Mesh] OR Sodium Glucose Transporter 2 Inhibitor OR SGLT-2 Inhibitor OR SGLT2 Inhibitor OR SGLT-2i OR sotagliflozin OR janagliflozin OR dapagliflozin OR canagliflozin OR empagliflozin OR ipragliflozin OR tofogliflozin OR ertugliflozin OR luseogliflozin OR sergliflozin OR licogliflozin OR remogliflozin OR bexagliflozin) AND (“Non-alcoholic Fatty Liver Disease”[Mesh] OR Non-alcoholic Fatty Liver Disease OR Nonalcoholic Fatty Liver OR Nonalcoholic Fatty Liver Disease OR Nonalcoholic Steatohepatitis OR Nonalcoholic Steatohepatitides OR Non-alcoholic Steatohepatitis OR Non-alcoholic Fatty Liver OR metabolic dysfunction-associated steatotic liver disease OR metabolic dysfunction-associated fatty liver disease OR NAFLD OR NASH OR NAFL OR MASLD OR MAFLD).

### Inclusion and exclusion criteria

Studies were considered for inclusion according to the Population Intervention Comparison Outcome Study design (PICOS) principles if they met all the following inclusion criteria: (1) the study design was a randomized controlled trial (RCT); (2) participants had MASLD (with or without T2DM); (3) the experimental group received SGLT-2 inhibitors, while the control group did not; and (4) the study reported outcomes related to hepatic fibrosis in patients with MASLD.

Articles that met one of the following exclusion criteria were excluded: (1) unavailable full text; (2) non-English language publications; (3) unavailable or missing statistical data; (4) Absence of target outcomes; (5) for the same cohort, only the most comprehensive and/or the most updated article was included; and (6) experimental groups received SGLT-2 inhibitors in combination with other drugs.

### Data extraction

Basic information of the included studies was collected independently by two investigators according to a pre-designed form. The extracted information included: (1) basic information of the studies (author, year, country, etc.); (2) participants’ characteristics (age, sex, comorbidities, etc.); (3) follow-up time; (4) study design and treatment protocol; and (5) liver fibrosis-related outcomes [e.g., Fib-4 index, liver stiffness measurement (LSM), and controlled attenuation parameter (CAP)].

The Fib-4 index is a brief and effective non-invasive method to evaluate liver fibrosis in patients with chronic liver disease. There is a positive correlation between its value and the progression of liver fibrosis (12). The formula for the Fib-4 index is as follows: Fib-4 = (Age [years] × AST [U/L])/(PLT [10^9^/L] × ALT^1/2^ [U/L]). In this study, an indicator was used as a routine, convenient tool for assessing the staging of liver fibrosis and the risk of disease progression.

### Aspartate aminotransferase, alanine aminotransferase, and platelets

Type 4 collagen 7s levels are an important component of the extracellular matrix, and their serum levels have been demonstrated to correlate closely with hepatic fibrogenic activity and the severity of fibrosis. This study used it as a biomarker to monitor the dynamic progression of liver fibrosis and to assess potential therapeutic responses (13).

The CAP is an ultrasound-based transient elastography measure of the liver used to assess hepatic steatosis. An increasing number of studies suggest that levels of steatosis are positively correlated with the severity of inflammatory damage and fibrosis in the liver. Therefore, it can be used to detect the degree of hepatic steatosis and predict liver fibrosis (14).

The LSM value is a FibroScan-based vibration-controlled transient elastography (VCTE) detector used to more objectively and accurately assess the extent of liver fibrosis. The higher the degree of liver fibrosis, the bigger the LSM value, which is a more intuitive method (15–17).

The NAFLD fibrosis score (NFS) is a non-invasive tool that can predict the severity of hepatic fibrosis in patients with NAFLD. A higher level indicates that the patient has a higher risk of liver fibrosis (18). The formula is as follows: NFS = −1.675 + 0.037 × age (years) + 0.094 × BMI (kg/m^2^) + 1.13 × diabetes mellitus (with = 1, without = 0) + 0.99 × AST/ALT ratio − 0.013 × PLT (×10^9^/L) − 0.66 × albumin (g/dL).

### Body mass index

#### Quality assessment

The methodological quality and risk of bias of the included randomized controlled trials were independently assessed by two investigators using the revised Cochrane risk of bias tool for randomized trials (RoB 1.0) (19). Any discrepancies in the assessment were resolved through discussion with a third senior investigator.

The tool evaluates the risk of bias across five domains:

1. Random sequence generation (selection bias).
2. Allocation concealment (selection bias).
3. Blinding of participants and personnel (performance bias).
4. Blinding of outcome assessment (detection bias).
5. Incomplete outcome data (attrition bias).
6. Selective reporting (reporting bias).
7. Other biases (e.g., baseline imbalance and source of funding).

For each domain, judgments of the risk of bias, such as “Low risk,” “High risk,” or “Unclear risk,” were made according to the criteria provided in the Cochrane Handbook.

### Statistical analysis

The mean difference (MD) and 95% confidence intervals (CIs) were used in the study to assess the relevance of SGLT-2 inhibitors for the liver fibrosis process in participants with MASLD. The MD and its 95% CI for each study were calculated from the post-intervention means, standard deviations, and sample sizes of the SGLT-2 inhibitors and control groups. A positive MD (MD >0) indicated a result favoring the control group, whereas a negative MD (MD <0) indicated a result favoring the experimental group. In addition, due to potential heterogeneity among the included studies—including different study designs, different populations, different types of SGLT-2 inhibitors, and different treatment durations—to increase the confidence of the results, the study chose to use random-effects models for the statistics, and the inverse-variance method was applied for pooling the effect sizes. The Cochrane *Q* chi-squared test was used to assess heterogeneity among studies, and the results were quantified using the *I*^2^ statistic. An *I*^2^ value of <30% was considered low heterogeneity; between 30 and 60% was considered moderate heterogeneity; and >60% was considered high heterogeneity. In addition, depending on the characteristics of the data being collected, subgroup analyses of the results were planned to explore the sources of heterogeneity. Additionally, a funnel plot was used to assess publication bias. To evaluate the stability of the results, sensitivity analysis was used. A two-sided *p*-value of <0.05 was considered statistically significant. RevMan version 5.3 was used for all of the abovementioned analyses.

## Results

### Search results and study characterization

A total of 1,532 relevant records were retrieved with the guidance of a predefined search strategy, and an additional 3 studies identified through other sources were added. After reviewing the literature and removing duplicate studies, 596 records remained. Among them, 540 records were excluded based on the screening of titles and abstracts, leaving 56 studies for full-text review. After full-text assessments, 40 studies were excluded for the following reasons: (1) not matching the protocol of this study; (2) non-RCTs; (3) outcomes of interest could not be identified; (4) outcome data were not available; and (5) already updated for publication.

A total of 16 RCTs (20–35) published from 2017 to 2024 involving more than 11,300 participants from all over the world were ultimately included. The majority of the studies included in the meta-analysis were from Asia [China (*n* = 2), Japan (*n* = 9), and Iran (*n* = 3)] and involved a large multicenter RCT (a study of 667 centers from 30 different countries). Among the included studies, 12 reported outcomes regarding the Fib-4 index, 7 reported outcomes regarding LSM, 6 reported CAP-associated outcome metrics, 4 reported outcomes regarding NFS, and 8 provided blood biochemical outcomes related to type 4 collagen 7s. Among the included studies, 2 applied the updated disease name MASLD. From the characteristics of the included cohorts, the majority of the studies included participants with T2MD, with the exception of only one study. Regarding the treatment options, a total of 6 different SGLT-2 inhibitors—namely dapagliflozin, empagliflozin, ipragliflozin, tofogliflozin, canagliflozin, and ertugliflozin—were chosen as experimental drugs and were compared to glimepiride, pioglitazone, metformin, and placebo, respectively. The duration of study follow-up ranged from 12 weeks to 240 weeks. Among the 16 included RCTs, the control groups received diverse interventions: placebo (*n* = 4), pioglitazone (*n* = 6), glimepiride (*n* = 2), standard care (*n* = 3), and sitagliptin (*n* = 1). For studies using pioglitazone as an active comparator, the daily doses were as follows: Yoneda et al. (25), Ito et al. (29), and Ito et al. (26) used 15–30 mg; Attaran et al. (30), Khaliq et al. (35), and Chehrehgosha et al. (34) used 30 mg. This heterogeneity in control treatments is an important consideration when interpreting the pooled effect estimates. The specific characteristics of the included studies are described in Table 1; Supplementary Table 1.

**Table 1:** Characteristics of the included RCTs in the meta-analysis.

References	Country	No. of participants	Participants’ characteristics	Intervention treatment	Controlled treatment	Follow-up (weeks)
Shimizu et al. (20)	Japan	57	T2DM + NAFLD	Dapagliflozin	Standard treatment	24 weeks
Takeshita et al. (21)	Japan	40	T2DM + NAFLD	Tofogliflozin	Glimepiride	48 weeks
Takahashi et al. (22)	Japan	50	T2DM + NAFLD	Ipragliflozin	Standard treatment	72 weeks
Shi et al. (23)	China	78	T2DM + NAFLD	Dapagliflozin	Standard treatment	24 weeks
Borisov et al. (24)	Multicenter study	10,131	T2DM + MASLD	Canagliflozin	Placebo	144 weeks
Yoneda et al. (25)	Japan	40	T2DM + NAFLD	Tofogliflozin	Pioglitazone	24 weeks
Ito et al. (26)	Japan	61	T2DM + MASLD	Ipragliflozin	Pioglitazone	240 weeks
Kinoshita et al. (27)	Japan	98	T2DM + NAFLD	Dapagliflozin	Glimepiride/pioglitazone	28 weeks
Tobita et al. (28)	Japan	22	T2DM + NAFLD	Dapagliflozin	Teneligliptin	12 weeks
Ito et al. (29)	Japan	66	T2DM + NAFLD	Ipragliflozin	Pioglitazone	24 weeks
Attaran et al. (30)	Iran	73	T2DM + NAFLD	Empagliflozin	Pioglitazone	24 weeks
Hiruma et al. (31)	Japan	113	T2DM + NAFLD	Empagliflozin	Sitagliptin	12 weeks
Weng et al. (32)	China	150	NAFLD	Dapagliflozin	Placebo	24 weeks
Taheri et al. (33)	Iran	90	T2DM + NAFLD	Empagliflozin	Placebo	24 weeks
Chehrehgosha et al. (34)	Iran	106	T2DM + NAFLD	Empagliflozin	Pioglitazone/placebo	24 weeks
Khaliq et al. (35)	Australia and New Zealand	180	T2DM + NAFLD	Ertugliflozin	Pioglitazone/placebo	24 weeks

RCTs, randomized controlled trials; No., number; T2DM, type 2 diabetes mellitus; NAFLD, non-alcoholic fatty liver disease; MASLD, metabolic-dysfunction-associated steatotic liver disease.

**Figure 1:**
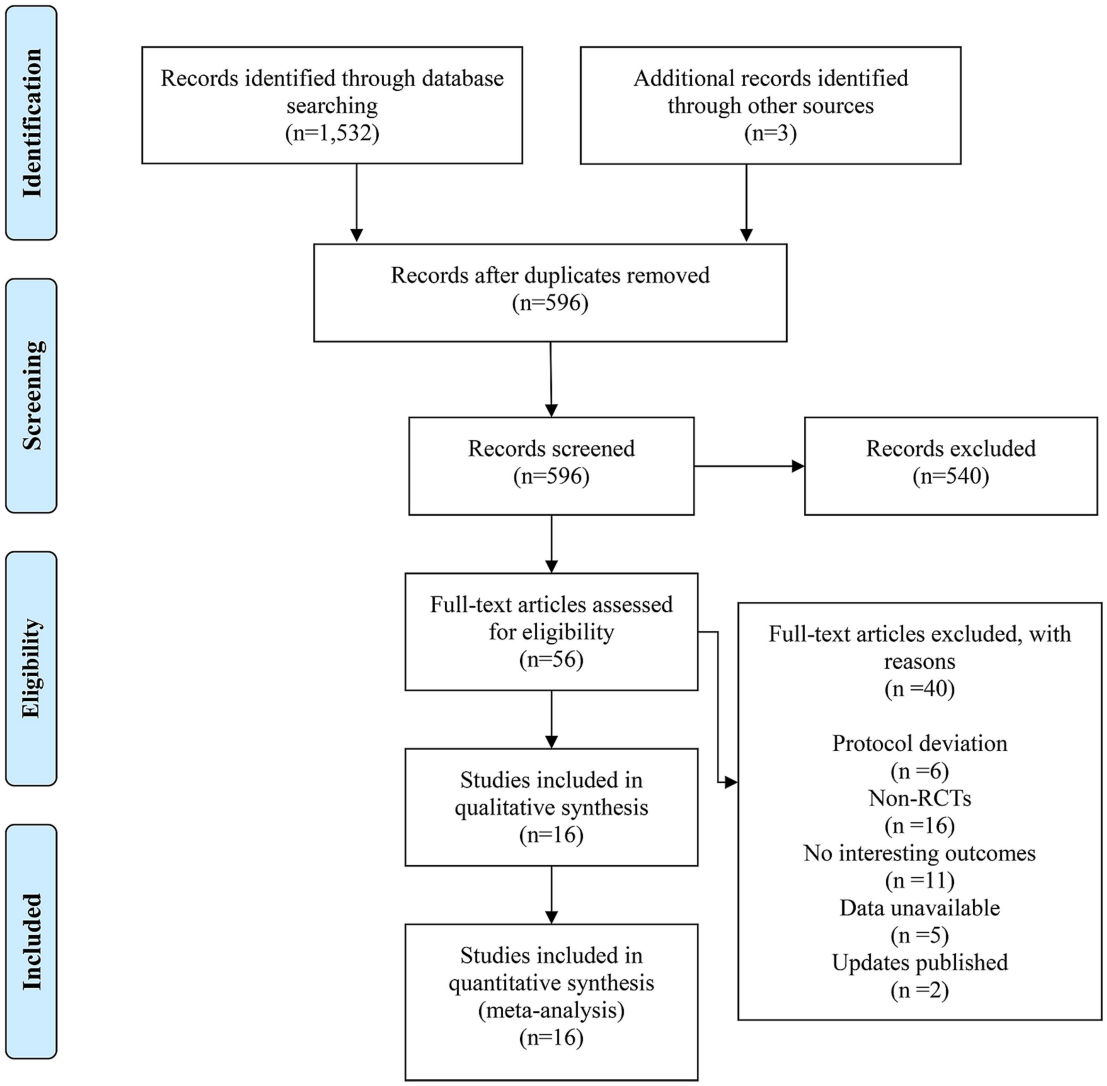
Flow diagram of literature selection.

The risk of bias for the included studies was assessed using the RoB 1.0 tool, and the results are illustrated in Supplementary Figures 1, 2. Generally, the included studies had low selection bias, but there were some concerns regarding blinding of participants and personnel, blinding of outcome assessment, and completeness of outcome data. In other words, there was a greater risk of performance bias, detection bias, and attrition bias (see Figure 1).

### Fib-4 index

**Figure 2:**
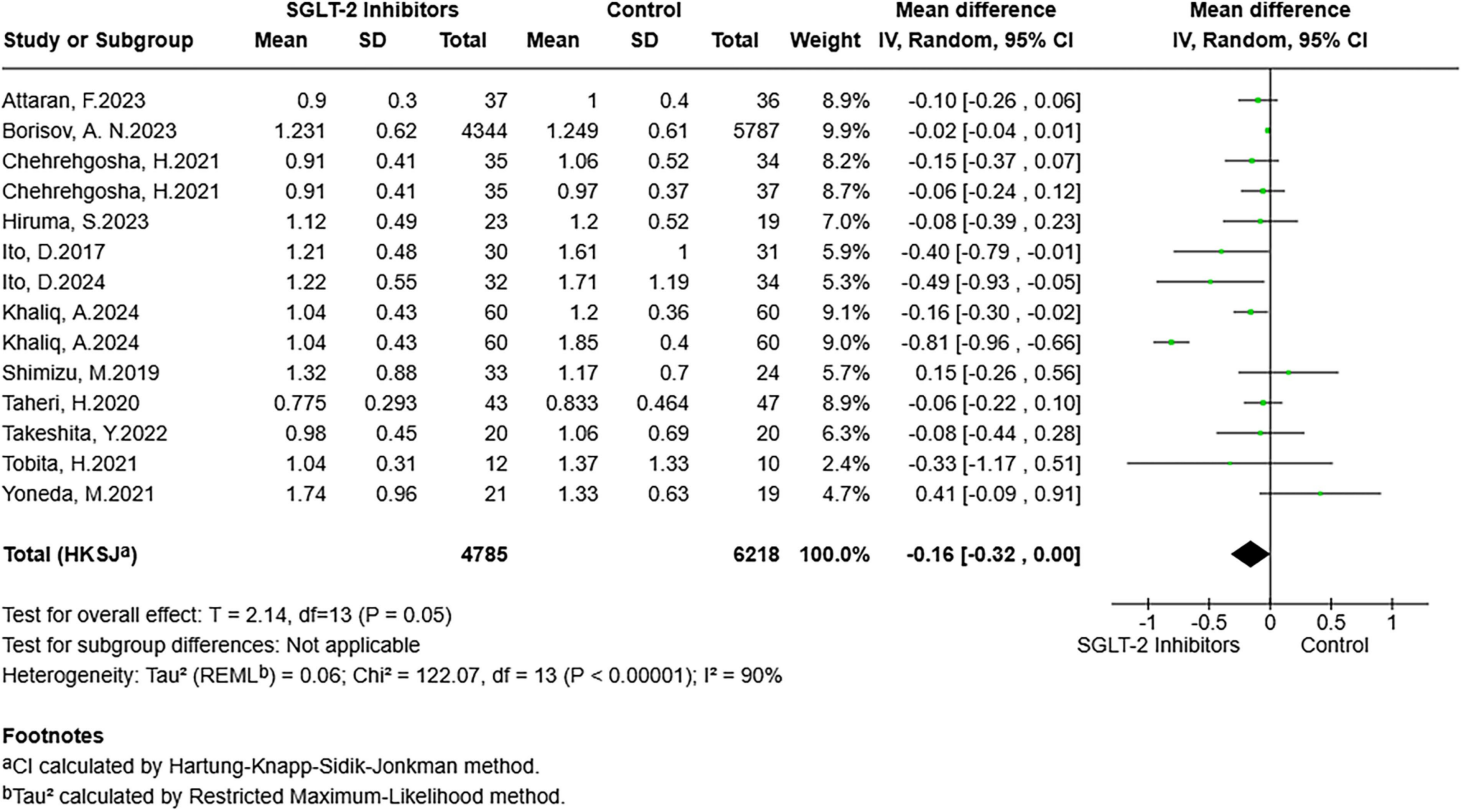
Forest plot of the association between SGLT-2 inhibitor exposure and the Fib-4 index in MASLD participants (Fib-4 index as a measure).

The pooled results of 12 studies showed a lower Fib-4 index in the SGLT-2 inhibitor-exposed group compared to the non-exposed group (MD = −0.16, 95% CI: −0.32 to 0.00, *p* = 0.05) (Figure 2). However, the chi-squared test assessment revealed higher heterogeneity (*I*^2^ = 90%). In addition, the funnel plot results of RCTs on the effect of SGLT-2 inhibitors on the participants’ Fib-4 index were roughly symmetrical, indicating no significant publication bias (Supplementary Figure 3). Finally, in the sensitivity analyses, each study was removed one by one, and the ultimate results remained stable. The subgroup analysis was used to explore the sources of heterogeneity (Table 2). In the subgroup of SGLT-2 inhibitor types, the analysis showed that empagliflozin-exposed participants had lower Fib-4 indices than non-exposed participants (MD = −0.09, 95% CI: −0.13 to −0.04, *p* = 0.006). Meanwhile, ipragliflozin-exposed participants also showed a lower Fib-4 index (MD = −0.44, 95% CI: −1.01 to 0.13, *p* = 0.06). Unfortunately, the above finding failed to reach statistical significance. On the contrary, for dapagliflozin, there was no statistically significant difference between the experimental and control groups (MD = 0.06, *p* = 0.81). In addition, for the subgroup of follow-up treatment duration, participants in the exposed group were observed to have relatively lower Fib-4 indices in the subgroup with <24 weeks of follow-up treatment compared with the control group (MD = −0.18, 95% CI −0.37 to 0.01, *p* = 0.07). However, the above results did not reach statistical significance. In the subgroup with >24 weeks of follow-up, there was no significant difference between the two groups (MD = −0.02, 95% CI −0.05 to 0.01, *p* = 0.18). Subgroups were analyzed according to the accompaniment of concomitant T2DM. The results of the study roughly suggested that those with T2DM using SGLT-2 inhibitors had a lower Fib-4 index than those not using SGLT-2 inhibitors; regrettably, the above results did not reach statistical significance (MD = −0.17, 95% CI: −0.36 to 0.02, *p* = 0.08). Meanwhile, there was no statistically significant difference in the results in the non-T2DM subgroup (MD = −0.07, 95% CI: −0.70 to 0.56, *p* = 0.40).

**Table 2:** Subgroup analysis of the association between SGLT-2 inhibitor exposure and the Fib-4 index.

Subgroup	No. of studies	MD	95% CI	*p*	*I*^2^ (%)
Types of SGLT-2 Inhibitors					
Dapagliflozin	2	0.06	[−2.35, 2.47]	0.81	1.0
Empagliflozin	4	−0.09	[−0.13, −0.04]	0.006	0
Ipragliflozin	2	−0.44	[−1.01, 0.13]	0.06	0
Follow-up time					
>24 weeks	3	−0.02	[−0.05, 0.01]	0.18	0
≤24 weeks	10	−0.18	[−0.37, 0.01]	0.07	84.0
T2DM					
Yes	10	−0.17	[−0.36, 0.02]	0.08	91.0
No	2	−0.07	[−0.70, 0.56]	0.40	0

SGLT-2 inhibitors, sodium–glucose transporter 2 inhibitors; T2DM, type 2 diabetes mellitus; No., number; MD, mean difference; 95% CI, 95% confidence intervals.

### Type 4 collagen 7s

**Figure 3:**
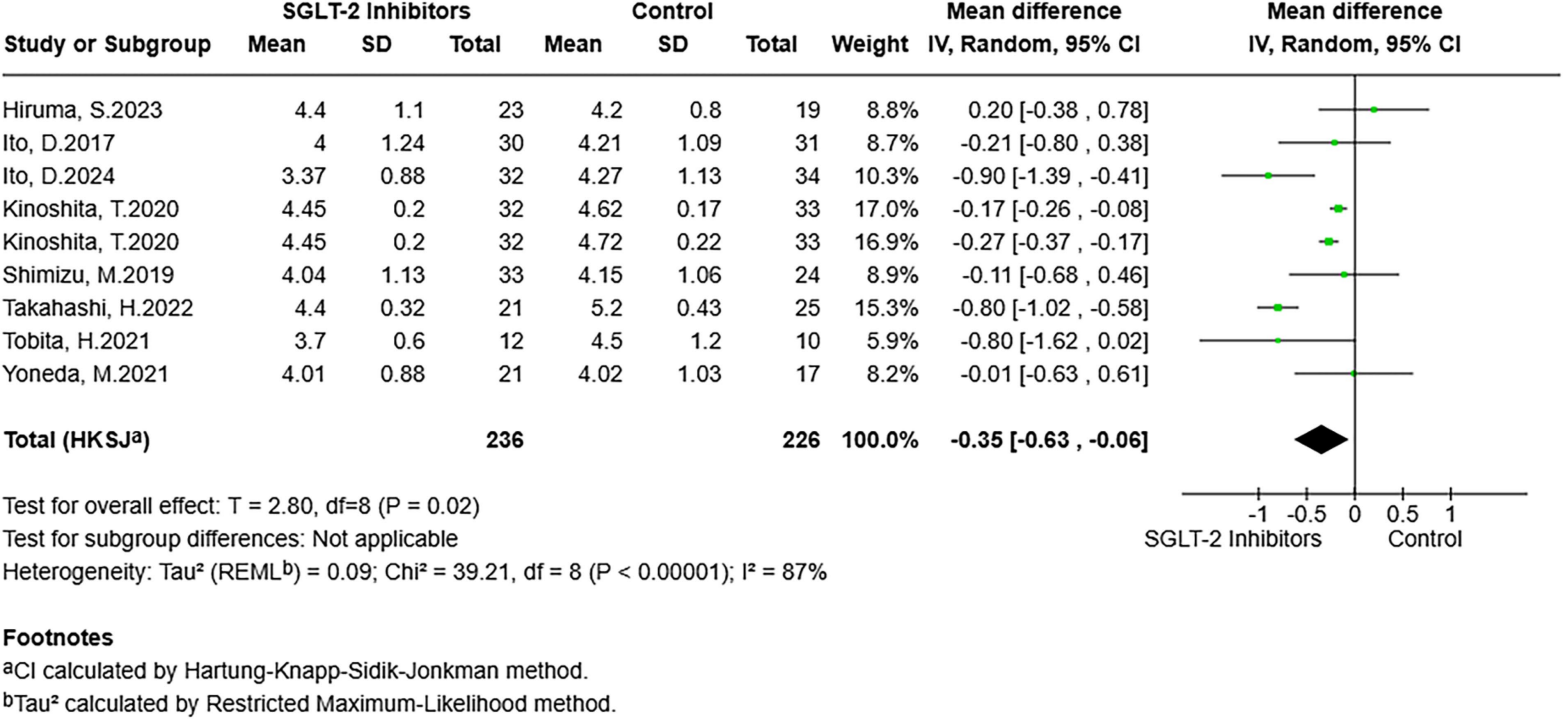
Forest plot of the association between SGLT-2 inhibitor exposure and the type 4 collagen 7s in MASLD participants.

The pooled results showed that the SGLT-2 inhibitor-exposed group had lower type 4 collagen 7s serum levels than the control group (MD = −0.35, 95% CI: −0.63 to −0.06, *p* = 0.02) (Figure 3). However, there was a high degree of heterogeneity (*I*^2^ = 87%). To explore the heterogeneity, a subgroup analysis was conducted (Table 3). In the dapagliflozin and ipragliflozin subgroups, participants in both experimental groups had lower levels of type 4 collagen 7s than those in the control group (dapagliflozin: MD = −0.22, 95% CI: −0.38 to −0.07, *p* = 0.02; ipragliflozin: MD = −0.71, 95% CI: −1.48 to 0.07, *p* = 0.06). It was regrettable that the latter did not reach statistical significance. For participants with T2DM, exposure to SGLT-2 could have lower type 4 collagen 7s levels compared with non-exposure (MD = −0.32, 95% CI: −0.63 to −0.01, *p* = 0.04).

**Table 3:** Subgroup analysis of the association between SGLT-2 inhibitor exposure and the type IV collagen 7s.

Subgroup	No. of studies	MD	95% CI	*p*	*I*^2^ (%)
Types of SGLT-2 inhibitors					
Dapagliflozin	3	−0.22	[−0.38, −0.07]	0.02	26.0
Ipragliflozin	3	−0.71	[−1.48, 0.07]	0.06	43.0
T2DM					
Yes	7	−0.32	[−0.63, −0.01]	0.04	89.0

SGLT-2 inhibitors, sodium–glucose transporter 2 inhibitors; T2DM, type 2 diabetes mellitus; No., number; MD, mean difference; 95% CI, 95% confidence intervals.

### Other indicators of liver fibrosis

**Figure 4:**
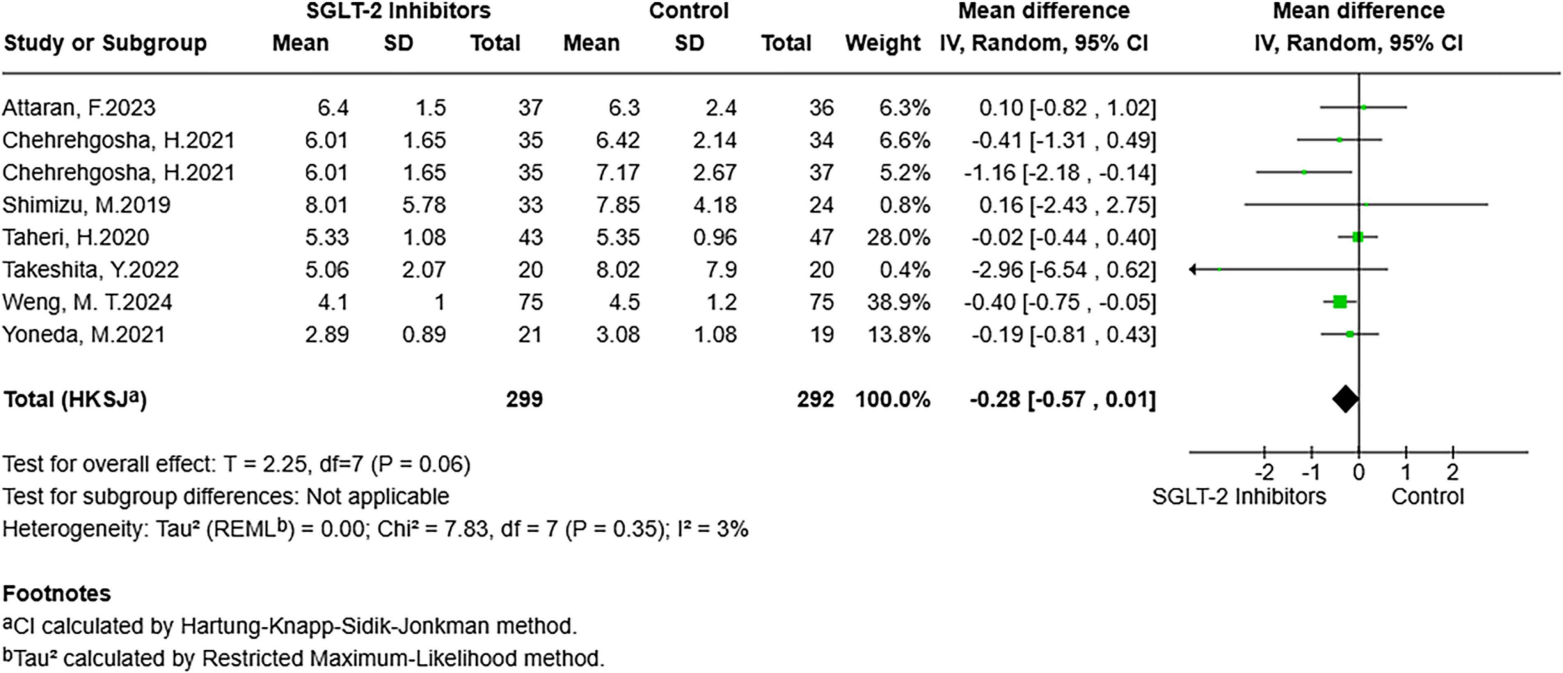
Forest plot of the association between SGLT-2 inhibitor exposure and the LSM in MASLD participants.

**Figure 5:**
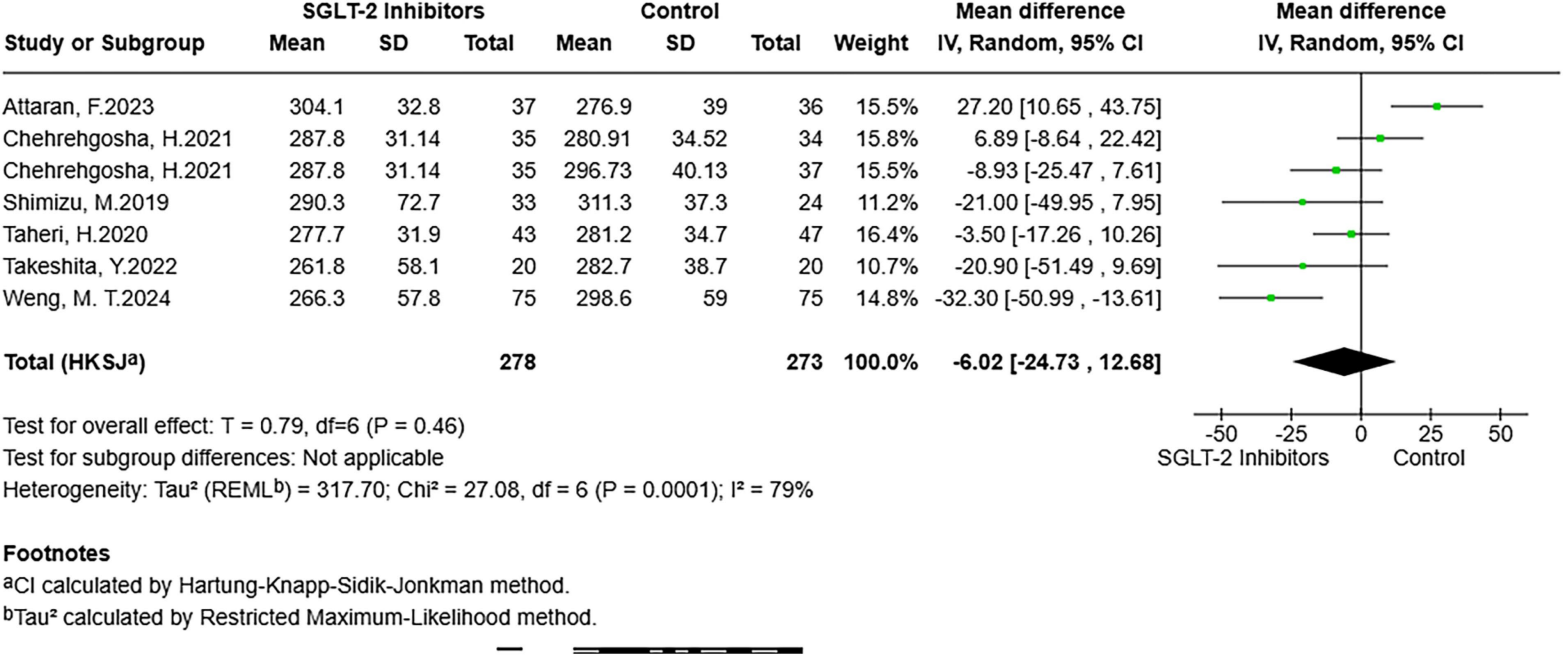
Forest plot of the association between SGLT-2 inhibitor exposure and the CAP in MASLD participants.

**Figure 6:**
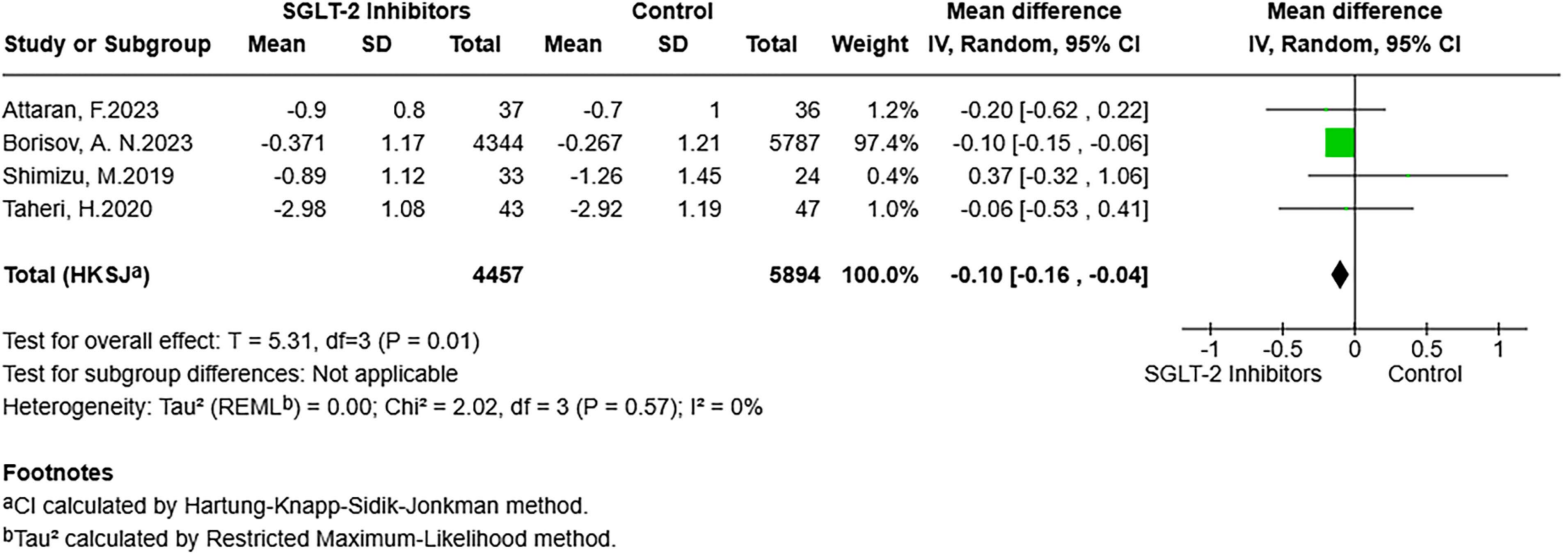
Forest plot of the association between SGLT-2 inhibitor exposure and the NFS in MASLD participants.

There were seven studies reporting results that included LSM. After pooling, despite the results not reaching statistical significance, a lower LSM was observed in participants exposed to SGLT-2 inhibitors compared to participants in the non-exposed group (MD = −0.28, 95% CI: −0.57 to 0.01, *p* = 0.06) (Figure 4). For CAP, the analysis of six studies that reported CAP outcomes was pooled. The meta-analysis suggested that CAP did not show statistically significant differences between exposure to SGLT-2 inhibitors and non-exposure to SGLT-2 inhibitors (MD = −6.02, 95% CI: −24.73 to 12.68, *p* = 0.46) (Figure 5). In addition, the NFS was included as an outcome of this study. After pooling studies with relevant data, the outcomes indicated the presence of lower NFS in the SGLT-2 inhibitor-exposed group compared to the non-exposed group (MD = −0.10, 95% CI: −0.16 to −0.04, *p* = 0.01) (Figure 6).

## Discussion

One currently popular drug for the treatment of T2DM is the SGLT-2 inhibitor. Previous studies have suggested that SGLT-2 inhibitors appear to have some therapeutic effects on MASLD. Therefore, to further explore the effect of SGLT-2 inhibitors on the progression of MASLD liver fibrosis, we performed an updated meta-analysis. Our study observed that SGLT-2 inhibitors have the potential to slow down liver fibrosis in MASLD, primarily in the following areas: Fib-4 index, NFS, and type 4 collagen 7s serum level.

NAFLD (currently known as MASLD) is known to be characterized by the excessive accumulation of triglycerides in the liver caused by a variety of reasons, which can eventually lead to a continued inflammatory state. Long-term inflammatory state of the liver and stimulation of liver damage can cause fibrosis and nodule formation, progressing to liver failure or cancer (36, 37).

At a microscopic level, disease progression may be possible in several ways. The massive accumulation of fat in the liver is the first change that occurs. The sterol regulatory element-binding protein 1 (SREBP1) is a regulator of hepatic lipogenesis genes (38, 39). In addition, specific fatty acid-binding proteins (FABPs) that are functional in transporting fatty acids, primarily the dominant FABP1, are able to affect the expression of peroxisome proliferator-activated receptor γ (PPARγ) by mediating its ligand transport into the hepatic cell nucleus (40). Overexpression of all of the above factors in the body can lead to massive production of lipids and the accumulation of harmful lipids (e.g., diglycerides), which can lead to the development of lipotoxicity and necroinflammation. Compromised normal fatty acid β-oxidation of lipids to alternative pathways of fatty acid oxidation (e.g., ω-oxidation) leads to persistent inflammation in the liver (41). The serology shows increased expression of inflammatory factors as well as necrosis and apoptosis of hepatocytes, which can eventually lead to fibrosis and even cirrhosis of the liver (36).

SGLT-2 inhibitors probably can work by regulating cellular substance metabolism and reducing fat accumulation. AMP-activated protein kinase (AMPK), an important regulator that can be activated by SGLT-2 inhibitors, prevents glucose uptake by decreasing cellular SGLT-2 expression (42, 43). On the other hand, SGLT-2 inhibitors have also been associated with the downregulation of lipid synthesis-related proteins and the upregulation of fatty acid oxidation-related genes in intrahepatic cells. For example, upregulation of PPAR-γ can trigger adipose synthesis from scratch, which, in turn, leads to the accumulation of lipids in hepatocytes (43). It was found that SGLT-2 inhibitors can downregulate the expression of PPAR-γ target genes, which can reduce liver weight and lipid accumulation. In addition, SGLT-2 inhibitors reduced hepatic triacylglycerol levels and ameliorated IR by reducing the formation of lipotoxic intermediates (44). Furthermore, it has also been found that mammalian target of rapamycin (mTOR) expression in the body can be reduced after using SGLT-2 inhibitors (45). The activity of SREBP1, which is directly regulated by mTOR signaling, can affect hepatic lipogenesis (38, 39, 46).

SGLT-2 inhibitors are also believed to play a role in reducing the level of inflammation in the liver. They reduce oxidative stress, inflammation levels, and apoptosis in the liver. An animal study found a decrease in intrahepatic cellular inflammatory factor levels (TNF-α, IL-1β, and IL-18) in dapagliflozin-treated rats and improved hepatic steatosis (47). In addition, myeloperoxidase (MPO), a chlorinating oxidant-generating enzyme, initiates acute inflammatory responses and promotes chronic inflammation through oxidant production (48, 49). It has been found that SGLT-2 inhibitors can reduce the production of MPO in hepatic tissues, which reduces the level of hepatic inflammation and improves the serum ALT level and the process of hepatic fibrosis (49).

In addition, hepatocyte fibrosis-related substances have been found to be regulated by SGLT-2 inhibitors (50). Transforming growth factor β (TGF-β) is considered to be a highly efficient inducer of liver fibrosis. It has been shown that SGLT-2 inhibitors can reduce hepatic TGF-β expression and thus slow down the process of liver fibrosis. Moreover, the antifibrotic effect of SGLT-2 inhibitors can also be exerted by reducing the expression of collagen 1a1, collagen 1a2, TGF-β, and smooth muscle actin (SMA) in the liver (51).

Second, SGLT-2 inhibitors, as popular hypoglycemic drugs, have been recommended by various guidelines as the drug of choice for the treatment of T2DM. They can improve the function of β-cells and sensitivity to insulin in patients with complicating T2DM, alleviate hepatic IR, and reduce the compensatory increase of insulin levels in the body. They slow down disease progression by improving the attenuation of lipid accumulation in the liver and hence slowing down disease progression (7). Our results based on the Fib-4 index and type 4 collagen 7s suggested that SGLT-2 inhibitors are beneficial in a subgroup of the population with T2DM and also support the above observations.

Although the majority of our outcomes support the efficacy of SGLT-2 inhibitors in delaying and controlling the progression of liver fibrosis in MASLD, there were still a few results that did not meet expectations. For indicators such as CAP, no beneficial effect of SGLT-2 inhibitors on the disease was observed. With respect to LSM, only a potential for SGLT-2 inhibitors to delay hepatic fibrosis was noted. This discrepancy may be explained by the distinct pathophysiological processes and technical characteristics captured by these different types of metrics.

Serological biomarkers, such as those integrated in the Fib-4 index (platelets, AST, and ALT) and circulating type 4 collagen 7s, are dynamic, systemic measures that reflect real-time hepatocyte injury, systemic inflammation, and extracellular matrix turnover. The primary metabolic benefits of SGLT-2 inhibitors—including improved insulin sensitivity, reduced hepatic lipotoxicity, and attenuated inflammation—may directly and rapidly influence these circulating factors (42, 44, 47). Consequently, improvements in these serological parameters might be detected earlier during intervention.

In contrast, LSM assessed by VCTE (e.g., FibroScan) quantifies the physical stiffness of liver tissue, which is a direct surrogate of established fibrosis architecture and scar deposition. Reversing this mechanical property likely requires prolonged tissue remodeling and collagen resorption, a process that may extend beyond the duration of most included trials (median follow-up ≤24 weeks) (52). Similarly, CAP estimates macroscopic hepatic fat content at a voxel level; as such, it may vary from less sensitive to subtle, early metabolic shifts within hepatocytes compared to systemic metabolic markers. It is also noteworthy that, in clinical practice, serum transaminase levels may remain within the normal range until advanced stages of disease, underscoring the complexity of interpreting any single biomarker (37). Therefore, LSM and CAP remain indispensable for staging established fibrosis and steatosis, while serological panels offer advantages for monitoring dynamic, short-term therapeutic responses.

The observed preferential improvement in serological indices in our analysis suggests that SGLT-2 inhibitors may exert their initial beneficial effects on pathways closely linked to hepatocellular health and systemic inflammation, which are promptly reflected in blood-based tests. Longer-term studies are warranted to determine whether these early serological improvements eventually translate into significant changes in liver stiffness, as measured by LSM.

In addition, we also observed that the trend of using SGLT-2 inhibitors in the short term (<24 weeks) still observed its beneficial effect on the progression of liver fibrosis. However, the results were not statistically significant in the subgroup with treatment follow-up >24 weeks. The reason for these differences may be due to the limited sample capacity, as the majority of the studies we included conducted short-term follow-up treatment (<24 weeks) and only three studies had long-term follow-up, which inevitably reduces statistical efficacy. Certainly, the presence of low adherence in long-term study subjects may also underestimate the efficacy of SGLT-2 inhibitors.

Our findings on the potential antifibrotic effects of SGLT2 inhibitors should be interpreted within the context of rapidly advancing therapeutic options for MASLD/MASH. Notably, resmetirom, a thyroid hormone receptor-β agonist, has recently been approved for the treatment of non-cirrhotic MASH with moderate to advanced liver fibrosis (stages F2–F3), based on histologically proven reversal of fibrosis and resolution of steatohepatitis. While resmetirom represents a targeted therapy for MASH, our meta-analysis suggests that SGLT2 inhibitors—a drug class with established benefits in glycemic control, cardiovascular, and renal outcomes—may also confer benefits on surrogate markers of liver fibrosis, particularly in the high-risk subgroup of patients with concomitant T2DM. This finding highlights the potential for different therapeutic strategies (direct hepatic-targeted vs. systemic metabolic agents) in the management of MASLD, depending on the patient’s predominant clinical phenotype and comorbidities. Future head-to-head or combination therapy studies are warranted to delineate their respective roles.

Compared with previous studies, the strength of our study focuses on the comprehensive analysis of the effect of SGLT-2 inhibitors on the progression of liver fibrosis in NAFLD (now known as MASLD) with the support of different rubrics. Second, we performed a further subgroup analysis to explore the sources of heterogeneity. Third, we also included the latest studies to increase the credibility of the results.

Despite its strengths, this meta-analysis has several important limitations that should be considered when interpreting the findings. First, there was significant heterogeneity across trials due to differences in the specific SGLT-2 inhibitor used, treatment duration, and control interventions (including active comparators such as pioglitazone and placebo). Although we used a random-effects model, this limits the precision of the pooled estimate. Second, all outcomes were assessed using non-invasive tests (NITs), without histological confirmation. The types of NITs used varied between studies, and detailed data on baseline fibrosis severity were unavailable, preventing the analysis of response by disease stage. Third, analytical depth was limited by data reporting. The majority of the studies did not provide the paired data needed to analyze within-group changes from baseline, thus our findings primarily reflect between-group differences at endpoint. Finally, some studies had risks of performance or detection bias. Nonetheless, the consistent direction of effect across serological markers provides a rationale for future, more definitive trials. Therefore, there is still a need for further well-designed, larger, multicenter RCTs to explore relevant aspects.

## Conclusion

Our meta-analysis suggests that SGLT-2 inhibitors may delay the progression of hepatic fibrosis in patients with MASLD, primarily reflected by improvements in the Fib-4 index, NFS, and serum type 4 collagen 7s levels. However, imaging indices such as LSM and CAP did not show significant differences.

Subgroup analyses suggested that the efficacy of different SGLT-2 inhibitors (e.g., empagliflozin and ipragliflozin) may vary. These drugs appeared more effective in short-term treatment (<24 weeks) and in patients with concomitant T2DM. However, some results were limited by higher heterogeneity and risk of bias. Further high-quality, large-sample, long-term follow-up studies are needed to validate their clinical value.

## Supplementary material

SUPPLEMENTARY FIGURE 1 Summarized RoB 1.0 of included studies.

SUPPLEMENTARY FIGURE 2 Summarized RoB 1.0 of included studies.

SUPPLEMENTARY FIGURE 3 Funnel plot of the association between SGLT-2 inhibitors exposure and Fib-4 index in MASLD participants.

## Glossary

NAFLD: Non-alcoholic fatty liver disease
IR: Insulin resistance
T2DM: Type 2 diabetes mellitus
MASLD: Metabolic dysfunction-associated steatohepatitis
SGLT-2: Sodium–glucose cotransporter-2
PRISMA: Preferred Reporting Items for Systematic Reviews and Meta-Analyses
RCTs: Randomized controlled trials
AST: Aspartate aminotransferase
ALT: Alanine aminotransferase
PLT: Platelets
LSM: Stiffness measurement
CAP: Controlled attenuation parameter
VCTE: Vibration-controlled transient elastography
NFS: NAFLD fibrosis score
BMI: Body mass index
MD: Mean difference
CI: Confidence intervals
SREBP1: Sterol regulatory element-binding protein 1
FABPs: Specific fatty acid-binding proteins
PPARγ: Peroxisome proliferator-activated receptor γ
AMPK: AMP-activated protein kinase
mTOR: Mammalian target of rapamycin
MPO: Myeloperoxidase
TGF-β: Transforming growth factor β
SMA: Smooth muscle actin
NITs: Non-invasive tests
MASH: Metabolic dysfunction-associated steatohepatitis
