# Supplementary figures and images for: Effect of SGLT-2 inhibitors on liver fibrosis progression in patients with MASLD: an updated meta-analysis based on RCTs
Source: Front Med (Lausanne). 2026 Jan 20;12:1667823. doi: 10.3389/fmed.2025.1667823 (PMC12864471; doi:10.3389/fmed.2025.1667823)

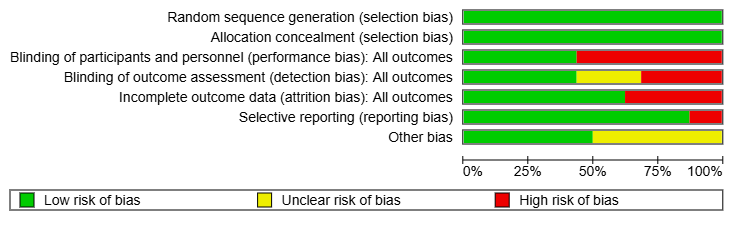

Supplement: SUPPLEMENTARY FIGURE 1 — Summarized RoB 1.0 of included studies. [file Image_1.PNG]

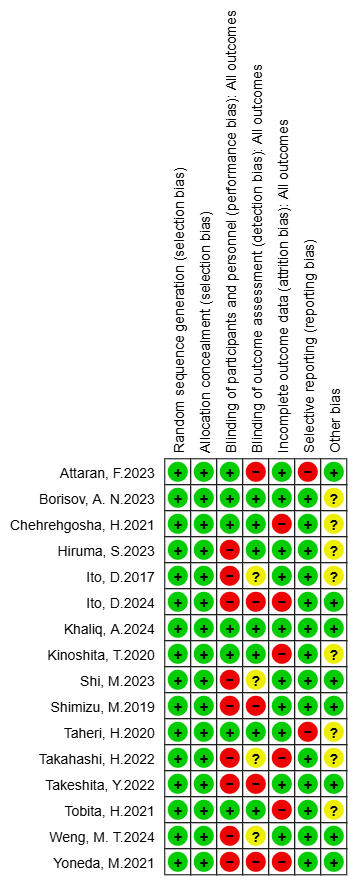

Supplement: SUPPLEMENTARY FIGURE 2 — Summarized RoB 1.0 of included studies. [file Image_2.PNG]

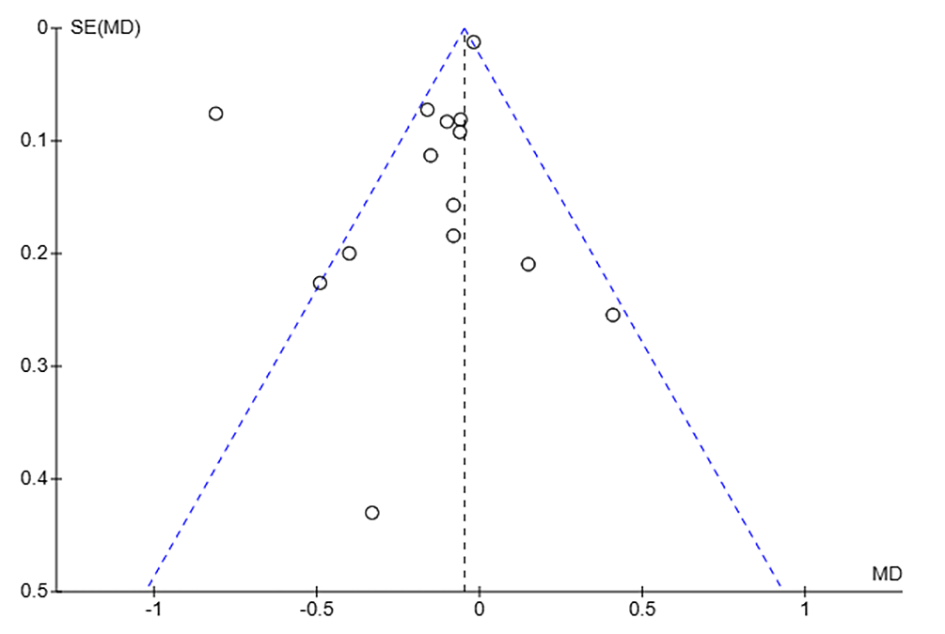

Supplement: SUPPLEMENTARY FIGURE 3 — Funnel plot of the association between SGLT-2 inhibitors exposure and Fib-4 index in MASLD participants. [file Image_3.TIF]
