# Supplementary material for: Effect of SGLT-2 inhibitors on liver fibrosis progression in patients with MASLD: an updated meta-analysis based on RCTs
Source: Front Med (Lausanne). 2026 Jan 20;12:1667823. doi: 10.3389/fmed.2025.1667823 (PMC12864471; doi:10.3389/fmed.2025.1667823)
Supplement: Supplementary file 4 [file Table_1.DOCX]

**Supplementary Table 1. Characteristics of included RCTs in the meta-analysis.**

| Author, year | Research code | No. of experimental group | No. of control group | DOI |
| --- | --- | --- | --- | --- |
| Shimizu, M. 2019 | UMIN000022155 | 33 | 24 | 10.1111/dom.13520 |
| Takeshita, Y. 2022 | NCT02649465/UMIN 000020544/jRCTs041180132 | 20 | 20 | 10.2337/dc21-2049 |
| Takahashi, H. 2022 | UMIN000015727/jRCTs071180069 | 24 | 26 | 10.1002/hep4.1696 |
| Shi, M. 2023 | ChiCTR2100054612 | 40 | 38 | 10.1016/j.jdiacomp.2023.108610 |
| Borisov, A. N. 2023 | NCT01032629+NCT01989754 | 4,344 | 5,787 | 10.1210/clinem/dgad249 |
| Yoneda, M. 2021 | jRCTs031180159 | 21 | 19 | 10.1136/bmjdrc-2020-001990 |
| Ito, D. 2024 | UMIN000040611 | 30 | 31 | 10.1111/jdi.14246 |
| Kinoshita, T. 2020 | UMIN 000021291 | 32 | 33 (pioglitazone)/33 (glimepiride) | 10.1111/jdi.13279 |
| Tobita, H. 2021 | UMIN000027304 | 12 | 10 | 10.3164/jcbn.20-129 |
| Ito, D. 2017 | UMIN000022651 | 32 | 34 | 10.2337/dc17-0518 |
| Attaran, F. 2023 | IRCT20190122042450N5 | 37 | 36 | 10.1186/s12876-023-02948-4 |
| Hiruma, S. 2023 | UMIN000026791 | 23 | 19 | 10.1111/dom.15006 |
| Weng, M. T. 2024 | NCT05308160 | 75 | 75 | 10.1007/s12072-024-10758-3 |
| Taheri, H. 2020 | IRCT20190122042450N1 | 43 | 47 | 10.1007/s12325-020-01498-5 |
| Chehrehgosha, H. 2021 | IRCT20190122042450N3 | 35 | 34 (pioglitazone)/37 (placebo) | 10.1007/s13300-021-01011-3 |
| Khaliq, A. 2024 | ACTRN12624000032550 | 60 | 60 (pioglitazone)/60 (placebo) | 10.1097/MD.0000000000040356 |

RCTs: randomized controlled trials; No.: number; DOI: digital object unique identifier.
